# Interaction between CDC6 and Tmod3 accelerates resistance to paclitaxel through focal adhesion assembly

**DOI:** 10.1038/s41392-025-02490-7

**Published:** 2025-12-04

**Authors:** Yue Liu, Huirui Wang, Jie Zhan, Jiabo Sun, Yan Sun, Xiaojie Fu, Dongxue Lv, Xiuyun Li, Ting Dong, Hongxiang Lou

**Affiliations:** 1 Key Laboratory of Natural Products & Chemical Biology, Ministry of Education, School of Pharmaceutical Sciences, Shandong University, Jinan, China; 2 Shandong Provincial Maternal and Child Health Care Hospital, Jinan, China; 3 Joint Research Institute of Medical & Pharmaceutical Sciences, Cheeloo Hospital of Shandong University, Jinan, China; 4 State Key Laboratory of Discovery and Utilization of Functional Components in Traditional Chinese Medicine, Jinan, China

**Keywords:** Target identification, Cancer therapy

## Abstract

The widespread clinical application of paclitaxel (PTX) in cancer treatment has been significantly limited by the emergence of drug resistance and the presence of drug-tolerant persister cells. To systematically identify key regulators of this resistance, we conducted a genome-wide CRISPR/Cas9 knockout screen, which revealed that cell division cycle 6 (CDC6) is a critical determinant of cell adhesion-mediated PTX resistance. Furthermore, our results illustrate that CDC6, an essential DNA replication licensing factor, functions through a pathway distinct from previously well-characterized resistance mechanisms. Genetic depletion of CDC6 considerably sensitizes cells, markedly increasing PTX-induced cell death. In addition to its established role in chromosome stability, CDC6 physically interacts with tropomodulin-3 (Tmod3) in the cytoplasmic compartment. This interaction enhances CDC6 protein stability and drives drug resistance phenotypes through the regulation of actin cytoskeleton remodeling and facilitating focal adhesion assembly. In addition, combination treatment with PTX and actin filament inhibitors synergistically enhanced the antitumor efficacy both in vitro and in vivo. Overall, our studies elucidate the mechanisms through which CDC6 functions as a key regulator of PTX resistance and provide a potential therapeutic strategy to increase PTX efficacy through the modulation of the cytoskeletal-adhesion axis.

## Introduction

Paclitaxel (PTX) is widely considered a standard chemotherapeutic agent for the treatment of various malignancies, including lung, breast, and ovarian cancers.^1^ Despite its established efficacy, the clinical application of PTX is greatly limited because of the development of acquired drug resistance and the emergence of drug-tolerant persisters.^2^ The current understanding of the mechanisms underlying PTX resistance has focused primarily on classical pathways. A well-characterized mechanism involves the overexpression of ATP-binding cassette (ABC) transporters, which facilitate drug efflux and reduce intracellular drug accumulation.^3^ Additionally, alterations in the expression or function of apoptosis-related proteins enable cancer cells to evade programmed cell death.^4^ Furthermore, structural or functional modifications in microtubule dynamics, including mutations in tubulin isoforms or changes in the expression of microtubule-associated proteins, can impair the binding affinity of PTX and subsequently diminish its cytotoxic effects.^5^ Recently, emerging evidence has suggested that cell adhesion contributes to the process of PTX resistance.^6–8^ However, the precise molecular pathways through which adhesion mediates resistance remain incompletely understood.

The mitotic process involves a series of morphological transformations that require dynamic disassembly and remodeling of focal adhesions. Studies have reported that cyclin-dependent kinase 1 (CDK1), in complex with Cyclin B, orchestrates adhesion restructuring during mitotic progression.^9^ Cell division cycle 6 (CDC6), an essential licensing factor that regulates the initiation of DNA replication during the G1 and S phases of the cell cycle,^10^ has increasingly been recognized as a significant oncogenic driver in a variety of cancer types.^11,12^ Furthermore, owing to its critical role in cell cycle progression and frequent dysregulation in tumors, CDC6 has also attracted attention as a promising diagnostic and prognostic biomarker in several cancer contexts.^13^ Although several studies have pointed to the potential role of CDC6 in drug resistance through regulating mitotic exit in human cells,^14,15^, its underlying involvement in adhesion-mediated resistance pathways remains uncharacterized.

The tropomodulin (Tmod) family represents a class of actin-binding proteins that exert precise regulatory control over the actin cytoskeleton. These proteins specifically cap the pointed ends of actin filaments (F-actin), thereby preventing actin monomers (G-actin) from associating and disassociating.^16^ In mammals, this family comprises four distinct isoforms that exhibit differential tissue expression patterns. Tmod1 and Tmod3 are broadly distributed across various tissues, whereas Tmod2 is enriched primarily in neurons, and Tmod4 is localized predominantly in skeletal muscles.^17^ Tmod3, in particular, has been demonstrated to participate in diverse fundamental biological processes, including the maintenance of actin filaments,^18^ the regulation of cell migration and lamellipodia formation,^19^ exocytotic processes,^20^ platelet development^21^ and the maturation of oocytes.^22^ In addition, the regulatory mechanisms of Tmod3 in oncogenesis have garnered increasing interest, particularly in light of its marked overexpression in hepatocellular carcinoma and pancreatic cancer.^23,24^ Recent studies revealed that asparagine endopeptidase (AEP) cleaves Tmod3 into two truncated fragments (tTmod3-N and tTmod3-C). The presence of these truncated forms is elevated in diverse tumor types and is correlated with a poor prognosis in high-grade gliomas. Functional analyses demonstrated that tTmod3-N and tTmod3-C regulate cancer cell proliferation and invasion via the nuclear SND1/RhoA pathway and cytoplasmic actin remodeling, respectively.^25^ Furthermore, Tmod3 promotes F-actin polymerization to facilitate the fusion of autophagosomes with lysosomes, a process that ultimately inhibits ferroptosis and confers cellular resistance to PD-1 antibody therapy.^26^ Nevertheless, it remains unclear whether Tmod3 is associated with paclitaxel resistance, and the potential relationship between Tmod3 and CDC6 has not been elucidated.

The development of CRISPR/Cas9 screening technology provides a promising strategy for identifying genes essential for the survival and proliferation of cancer cells, as well as genes contributing to drug resistance.^27–29^ In the present study, through a genome-wide CRISPR/Cas9 knockout screening, we identified CDC6 as a critical regulator of paclitaxel resistance. Notably, CDC6 functions through a mechanism independent of previously reported resistance-associated genes, including tubulin beta 3 class III (TUBB3) and ATP binding cassette subfamily B member 1 (ABCB1).^30^ Mechanistically, Tmod3 interacts directly with CDC6, and Tmod3 deficiency, along with Tomd1 or CDC6 deficiency, results in a severe disruption of actin filaments and disassembly of focal adhesions. Additionally, downregulating CDC6 increased the sensitivity of cells to paclitaxel both in vitro and in vivo. In conclusion, our research revealed a previously unrecognized mechanism of paclitaxel resistance and provided an alternative way to target CDC6 to overcome chemoresistance.

## Results

### CRISPR library screening identifies CDC6 as a critical regulator of paclitaxel resistance

**Fig. 1:**
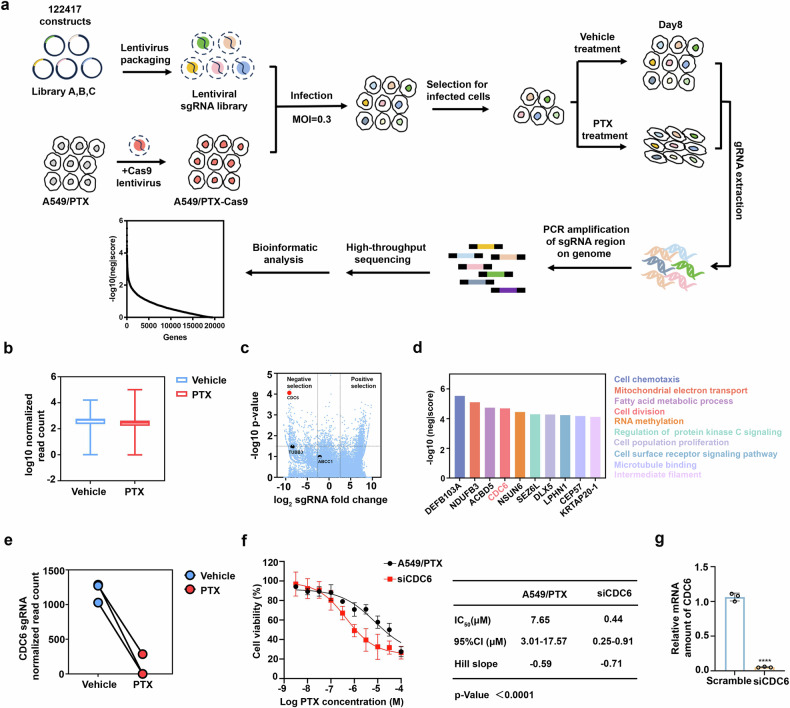
CRISPR library screening identifies CDC6 as a critical regulator of paclitaxel resistance. **a** Workflow of genome-wide CRISPR/Cas9 knockout library screening. We packed a human genome-wide CRISPR/Cas9 knockout library that contains 122,417 sgRNAs into lentiviral particles and transduced them into A549/PTX cell lines with stable overexpression of Cas9. Puromycin was used to select the transduced cells, which were then cultured in fresh medium containing paclitaxel for an additional 8 days. The genomic DNA was then extracted and amplified via PCR. After DNA gel extraction, the products were subjected to high-throughput sequencing to identify potential genes related to drug resistance. **b** Normalized read counts of all the sgRNAs in the vehicle- and paclitaxel-treated samples. **c** sgRNAs that were selected for paclitaxel treatment. **d** The top ten negatively identified genes in the screening. **e** The normalized read count of CDC6 suggested that the number of sgRNAs targeting CDC6 was significantly reduced in cells treated with paclitaxel. **f** The cytotoxicity of paclitaxel in A549/PTX wild-type cells and CDC6-knockdown cells. **g** Quantitative real-time PCR analysis was used to detect the mRNA level of CDC6 after A549/PTX cells were transfected with small-interfering RNA (n = 3; ****P < 0.0001)

To identify crucial biological processes related to paclitaxel resistance, we performed RNA- sequencing (RNA-seq) to analyze differential gene expression profiles between sensitive and resistant A549 cells. The analysis revealed 2,418 significantly dysregulated genes (|log2FC| >1.0, P < 0.05), with Gene Ontology enrichment revealing predominant involvement in cell adhesion-related pathways (Supplementary Fig. 1a–c). These results suggested that increased focal adhesions might directly contribute to paclitaxel resistance. CRISPR/Cas9 technology enables genome-scale, high-precision functional gene screening, providing a revolutionary tool for systematically dissecting the genetic basis of paclitaxel resistance. Therefore, we conducted a genome-wide CRISPR-Cas9 knockout library screening to systematically delineate genetic determinants of paclitaxel resistance (Fig. 1a).

Stable cell lines overexpressing Cas9 were constructed from A549 paclitaxel-resistant cell lines (A549/PTX), which presented increased IC_50_ values against paclitaxel (Supplementary Fig. 1d, e). Following lentiviral library transduction and puromycin selection, genome-edited cell pools were subjected to vehicle or paclitaxel treatment for 8 days. High-throughput sequencing analysis revealed the selective depletion of sgRNAs targeting genes essential for survival under paclitaxel pressure, with comparative read count distributions confirming screening validity (Fig. 1b). Unexpectedly, although previously characterized resistance-associated genes such as TUBB3 and ABCC1 were identified in our screening, their lower rankings prompted us to prioritize top-ranked candidates for subsequent mechanistic investigation (Fig. 1c). The functional annotation of the top 10 genes highlighted roles in mitochondrial electron transport, fatty acid metabolism, cell cycle regulation, RNA methylation and other life processes (Fig. 1d). Preliminary validation studies demonstrated that siRNA-mediated silencing of NDUFB3 and ACBD5 failed to sensitize A549/PTX cells to paclitaxel (Supplementary Fig. 1f, g). Given the intrinsic connection between cell cycle regulation and adhesion dynamics, we focused our attention on CDC6, an important gene that is involved in cell division. Further analysis revealed that the number of sgRNAs targeting CDC6 significantly decreased in the paclitaxel-treated group, suggesting that the loss of CDC6 might improve the therapeutic efficacy of paclitaxel (Fig. 1e). Additionally, the MTT assay demonstrated that depleting CDC6 markedly enhanced the cytotoxic activity of paclitaxel in PTX-resistant cells (Fig. 1f, g). In conclusion, our findings indicate that CDC6 plays a vital role in the development of paclitaxel resistance.

### Inhibition of CDC6 sensitizes A549/PTX cells to paclitaxel treatment

**Fig. 2:**
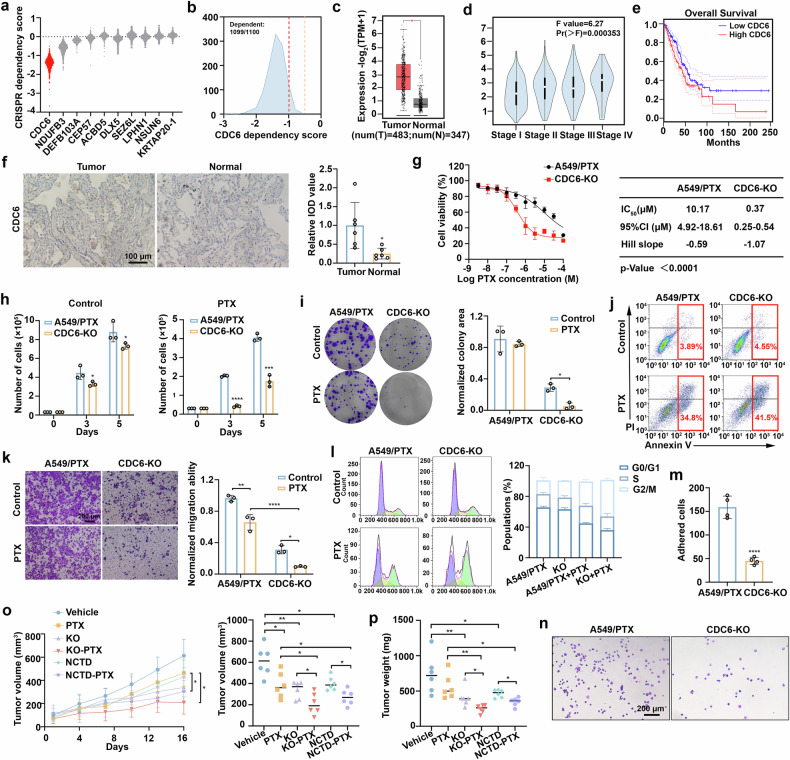
Inhibition of CDC6 sensitizes A549/PTX cells to paclitaxel treatment. **a** Violin plot showing the CRISPR dependency scores of the top ten genes related to drug resistance via DepMap data. **b** Smoothed histogram showing the CDC6 dependency score distribution among cancer cell lines from the CRISPR–spCas9 DepMap data. **c** mRNA expression levels of CDC6 in human lung adenocarcinoma tissues and normal lung tissues. The data are shown as box-and-whisker plots (* P < 0.05). The images were obtained from the DEPIA database. **d** CDC6 expression in the LUAD pathological stage plot (P = 0.000353). The image was obtained from the DEPIA database. **e** Kaplan–Meier plot of LUAD patients with different expression levels of CDC6. The image was obtained from the DEPIA database (n (high)=239, n (low)=239, Logrank p = 0.0049). **f** IHC examination of CDC6 expression in lung cancer tissue and adjacent normal tissue (n = 6; *P < 0.05). Scale bar, 100 µm. **g** A549/PTX cells and CDC6-KO cells were exposed to the indicated doses of paclitaxel for 72 h. **h** Numbers of A549/PTX cells and CDC6-KO cells after paclitaxel (300 nM) treatment for 3 and 5 days (n = 3; *P < 0.05, ***P < 0.001, ****P < 0.0001). **i** Colony formation assay in cells treated with paclitaxel (n = 3; *P < 0.05). **j** Knockout of CDC6 increased paclitaxel (600 nM)-induced apoptosis. (k) Transwell migration experiment in cells treated with paclitaxel (n = 3; *P < 0.05, **P < 0.01, ****P < 0.0001). Scale bar, 200 µm. **l** Cell cycle population after treatment with paclitaxel (50 nM) for 24 h. **m**, **n** A fibronectin adhesion assay was performed to test focal adhesion (FA) formation for 1 h (n = 4; ****P < 0.0001). Scale bar, 200 µm. **o**, **p** The tumor volumes and tumor weights are shown (n = 6 mice/group; *P < 0.05, **P < 0.01). The data are presented as the means ± SDs

To determine whether CDC6 functions as a cancer-related gene, we utilized data from the Cancer Dependency Map (DepMap), which includes genome-wide CRISPR loss-of-function screens across multiple cancer cell lines. The results revealed that the survival of multiple cancer cell lines was most dependent on CDC6 (Fig. 2a). In addition, CDC6 was classified as a cancer-dependent gene in 1099 out of the 1100 cell lines analyzed (Fig. 2b). The mRNA expression of CDC6 was considerably elevated in many tumor tissues, such as lung adenocarcinoma (LUAD), compared with normal tissues (Fig. 2c, Supplementary Fig. 2a). Importantly, CDC6 expression was positively correlated with advanced pathological stage in LUAD patients (Fig. 2d). High CDC6 expression was associated with poorer overall survival than low CDC6 expression was (Fig. 2e). Immunohistochemical (IHC) analysis further confirmed elevated CDC6 expression in human lung cancer tissues (Fig. 2f). Collectively, these findings indicate that CDC6 plays a critical role in human cancer.

To elucidate the relationship between CDC6 and paclitaxel resistance, we treated A549 cells with paclitaxel for 7 days, which resulted in significant upregulation of CDC6 (Supplementary Fig. 2b). CRISPR-mediated CDC6 knockout (KO) in PTX-resistant A549/PTX cells (Supplementary Fig. 2c) modestly reduced baseline proliferation but dramatically potentiated paclitaxel-induced cytotoxicity (Fig. 2g, h). Conversely, CDC6 overexpression conferred resistance to paclitaxel in parental A549 cells (Supplementary Fig. 2d, e). The results of functional assays revealed that CDC6 ablation significantly impaired colony formation (Fig. 2i) and increased apoptotic rates (Fig. 2j) while attenuating both cell migration capacity (Fig. 2k) and cell cycle progression (Fig. 2l). Mechanistically, CDC6 deficiency disrupted fibronectin-mediated cell adhesion (Fig. 2m, n), suggesting its critical role in adhesion-dependent chemoresistance. In vivo validation revealed markedly greater paclitaxel sensitivity in CDC6-KO xenografts than in control xenografts (Fig. 2o, p and Supplementary Fig. 2f, g). The clinically utilized antitumor drug norcantharidin (NCTD) has been established as a CDC6 inhibitor,^31^ which is consistent with our experimental findings (Supplementary Fig. 2h). Therefore, we tested the therapeutic effect of paclitaxel in combination with NCTD in vitro (Supplementary Fig. 2i–k) and in vivo. Compared with PTX alone, the combination treatment notably decreased the tumor volume and tumor weight (Fig. 2o, p and Supplementary Fig. 2l). In conclusion, these findings confirmed a strong association between CDC6 and paclitaxel resistance.

### Depletion of CDC6 improves paclitaxel efficacy partially by inducing multipolar divisions

**Fig. 3:**
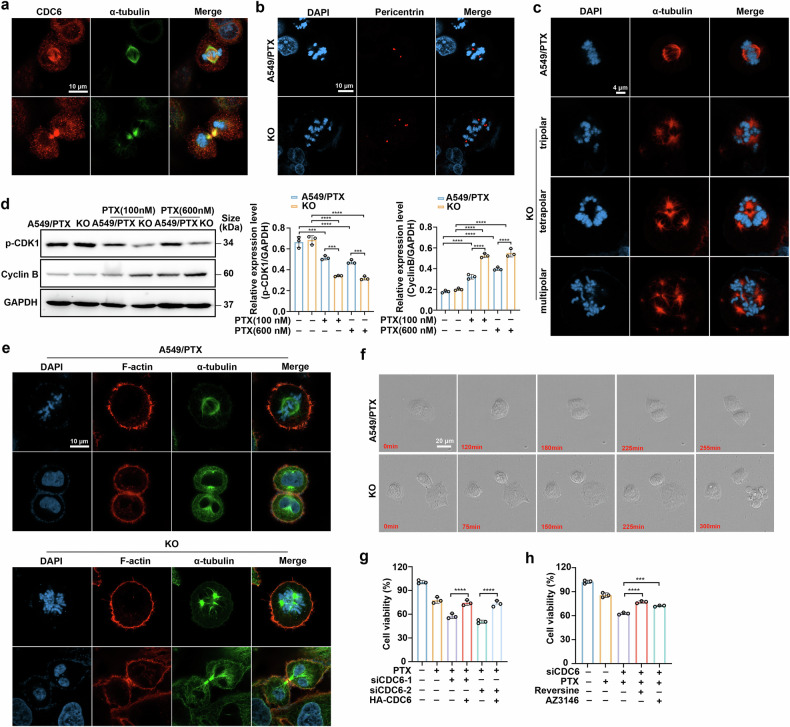
Depletion of CDC6 improves paclitaxel efficacy partially by inducing multipolar divisions. **a** Immunofluorescence staining of cells with a CDC6 (red) antibody, an α-tubulin (green) antibody and DAPI (blue). Scale bar, 10 µm. **b** Immunofluorescence staining of cells with a pericentrin (red) antibody and DAPI (blue). Scale bar, 10 µm. **c** Immunofluorescence staining of cells with an α-tubulin (red) antibody and DAPI (blue). The pictures represent mitotic spindles with the indicated number of poles. Scale bar, 4 µm. **d** Western blotting analysis of the expression of p-CDK1 and Cyclin B after treatment with the indicated doses of paclitaxel (n = 3; ***P < 0.001, ****P < 0.0001). **e** Immunofluorescence staining of wide-type and CDC6-KO cells with F-actin (red), α-tubulin (green) antibody and DAPI (blue). Scale bar, 10 µm. **f** Cells were cultured in a 96-well plate, and images were captured via time-lapse microscopy. Scale bar, 20 µm. **g** Viability of A549/PTX cells following treatment with siCDC6 oligo, HA-tagged CDC6 and paclitaxel (n = 3; ****P < 0.0001). **h** Viability of A549/PTX cells following treatment with siCDC6 oligo, reversine (0.5 μM), AZ3146 (1 μM) and paclitaxel (n = 3; ***P < 0.001, ****P < 0.0001). The data are presented as the means ± SDs

Considering the important role of CDC6 in regulating cell mitosis and microtubule organization, we detected the distribution of CDC6 and found that CDC6 colocalized with α-tubulin during the process of mitosis (Fig. 3a). According to previous research, deficiency in CDC6 may lead to centrosome overduplication.^32^ Therefore, we performed immunofluorescence experiments, and the results consistently revealed two normal centrosomes in wild-type cells, whereas multiple abnormal centrosomes were found in CDC6-depleted cells (Fig. 3b and Supplementary Fig. 3a). In addition, mitotic spindle analysis further demonstrated increased multipolar spindle formation following CDC6 ablation compared with that in control cells (Fig. 3c and Supplementary Fig. 3b). These findings collectively suggest that CDC6 depletion induces chromosomal instability through aberrant mitotic machinery, which was previously shown to increase paclitaxel efficacy.^33^

Paclitaxel-treated cancer cells usually experience prolonged mitotic arrest, ultimately leading to cell death. However, paclitaxel-resistant cells may evade mitotic arrest through a process known as mitotic adaptation or slippage.^15^ Specifically, the slow degradation of Cyclin B and the subsequent activation of CDK1 in cells result in the activation of separase, an enzyme that resolves sister chromatid cohesion, which ultimately leads to premature exit from mitosis.^34^ As CDC6 has been identified as a key component in the mitotic CDK1 activation cascade,^14^ we examined its regulatory effects on mitotic exit. CDC6 depletion significantly reduced p-CDK1 levels and increased the expression of Cyclin B in the presence of paclitaxel (Fig. 3d), indicating an impediment to premature mitotic exit. Live-cell imaging confirmed that CDC6-deficient cells presented profound mitotic defects including multinucleation (Fig. 3e) and catastrophic mitotic death (Fig. 3f). Additionally, rescue experiments in CDC6-knockdown cells demonstrated that the therapeutic efficacy of paclitaxel was significantly attenuated (Fig. 3g). Reversine and AZ3146, two kinase monopolar spindle 1 (Msp1) inhibitors, are reported to reduce the incidence of multipolar spindles in the late stages of mitosis.^33^ The application of reversine or AZ3146 could partially reverse the effects of paclitaxel in CDC6-knockdown cells (Fig. 3h), suggesting that the sensitivity to paclitaxel caused by CDC6 deprivation is partially due to multipolar divisions and that other mechanisms remain to be discovered.

### Tmod3 interacts with CDC6 and is associated with paclitaxel resistance

**Fig. 4:**
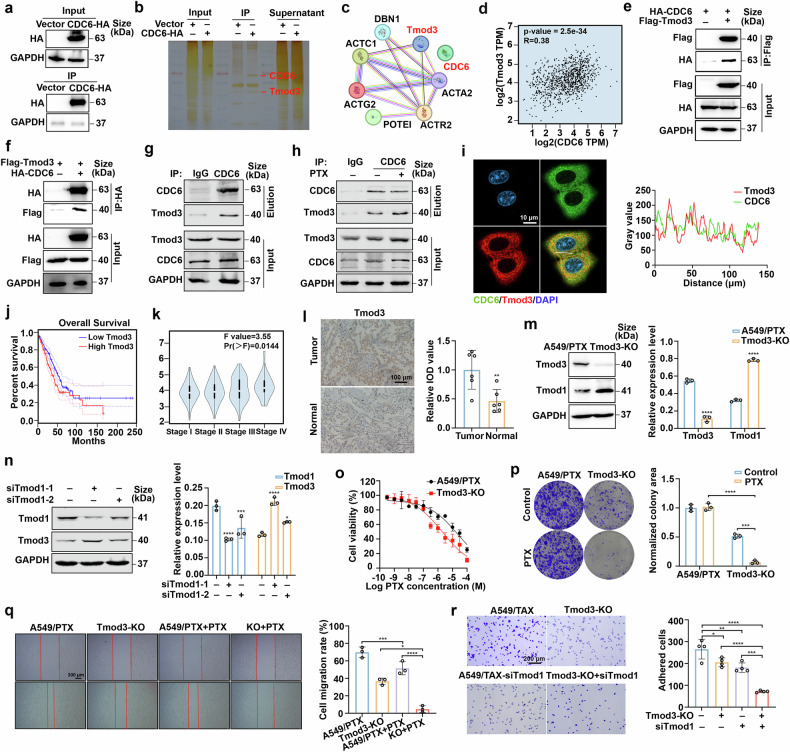
Tmod3 interacts with CDC6 and is associated with paclitaxel resistance. **a**, **b** 293 T cells were transfected with CDC6 tagged with HA for 48 h. The cells were subsequently lysed and immunoprecipitated with anti-HA magnetic beads. **c** STRING analysis of the potential proteins that may interact with CDC6. **d** Scatter plot showing linear regression between the mRNA expression of Tmod3 (*y*-axis) and that of CDC6 (*x*-axis). **e**, **f** 293 T cells were transfected with CDC6 tagged with HA and Tmod3 tagged with Flag for 48 h. The interactions between CDC6 and Tmod3 were analyzed via western blotting. **g** A co-IP assay was performed in A549 cells, and the interactions between CDC6 and Tmod3 were analyzed via western blotting. **h** A co-IP assay was performed in A549 cells treated with paclitaxel for 10 days and the interactions between CDC6 and Tmod3 were analyzed via western blotting. **i** Confocal images showing the colocalization of CDC6 (green) and Tmod3 (red). Scale bar, 10 μm. **j** Kaplan–Meier plot of LUAd patients with high or low expression of Tmod3. The image was obtained from the DEPIA database (n (high) = 239, n (low) = 238, Logrank p = 0.011). **k** Tmod3 expression in the LUAD pathological stage plot (P = 0.0144). The image was obtained from the DEPIA database. **l** IHC examination of Tmod3 expression in normal tissue and lung cancer tissue (n = 6; **P < 0.01). Scale bar, 100 µm. **m** Changes in Tmod1 expression in Tmod3-deficient cells (n = 3; ****P < 0.0001). **n** Changes in Tmod3 expression in Tmod1-deficient A549/PTX cells (n = 3; *P < 0.05, ****P < 0.0001). **o** Cells were exposed to the indicated doses of paclitaxel for 72 h. Cell viability was evaluated via the MTT assay. **p** A clonogenic assay was conducted in cells treated with paclitaxel (n = 3; ***P < 0.001, ****P < 0.0001). **q** A wound healing assay was conducted after the cells were exposed to paclitaxel (n = 3; *P < 0.05, ***P < 0.001, ****P < 0.0001). Scale bar, 300 μm. **r** A fibronectin adhesion assay was performed to test FA formation for 1 h (n = 4; *P < 0.05, **P < 0.01, ***P < 0.001, ****P < 0.0001). Scale bar, 200 µm. The data are presented as the means ± SDs

To elucidate the mechanisms by which CDC6 promotes paclitaxel resistance through regulating focal adhesion, we tagged CDC6 with HA and performed an immunoprecipitation (IP) assay. A distinctive protein band at approximately 40–55 kDa was observed and analyzed via liquid chromatography-tandem mass spectrometry (LC/MS). Considering the role of Tmod3 in actin remodeling and focal adhesion, we identified Tmod3 as a candidate protein that interacts with CDC6 (Fig. 4a–c). Tmod3 is an actin-binding protein that is widely expressed in human tissues and contributes to various life activities, including maintaining cytoskeletal stress fibers, regulating pseudopodia, maturing oocytes, and facilitating cell secretion.^35^ A recent study indicated that high Tmod3 expression is notably involved in the occurrence and progression of cancer.^23^ Gene expression profiling interactive analysis (GEPIA) revealed a positive relationship between the mRNA expression levels of Tmod3 and CDC6 (Fig. 4d). Additionally, complementary co-IP experiments in HEK293T cells confirmed the physical interaction between CDC6 and Tmod3 (Fig. 4e, f), which was also proven in A549 cells (Fig. 4g). We observed a significant increase in the CDC6-Tmod3 interaction when A549 cells were continuously treated with paclitaxel to simulate the drug resistance process (Fig. 4h). Furthermore, immunofluorescence staining revealed the colocalization of CDC6 and Tmod3 in both parental A549 cells and paclitaxel-resistant A549/PTX cells (Fig. 4i and Supplementary Fig. 4a). To provide direct evidence of this interaction in living cells, a bimolecular fluorescence complementation (BiFC) assay was conducted, which further confirmed that CDC6 and Tmod3 directly interact (Supplementary Fig. 4b).

We next sought to identify the role of Tmod3 in paclitaxel resistance. Clinical correlation analysis revealed the prognostic significance of Tmod3, with reduced overall survival (Fig. 4j), high expression correlating with advanced LUAD staging (Fig. 4k), and elevated protein levels in patient tumors and A549/PTX cells (Fig. 4l and Supplementary Fig. 4c, d). Surprisingly, compensatory Tmod1 upregulation was observed in Tmod3 knockout cells; conversely, Tmod3 was upregulated in Tmod1-depleted models (Fig. 4m, n and Supplementary Fig. 4e). Notably, this reciprocal regulation was conserved in A549 cells (Supplementary Fig. 4f, g). Therefore, we performed IP experiments, and the results confirmed the interaction between CDC6 and Tmod1 (Supplementary Fig. 4h), indicating that both Tmod1 and Tmod3 may play cooperative roles in paclitaxel resistance. Functional analysis revealed that depletion of Tmod3 substantially enhanced cellular sensitivity to paclitaxel (Fig. 4o) and effectively suppressed colony formation (Fig. 4p), cell migration (Fig. 4q) and cell adhesion (Fig. 4r), whereas simultaneously knocking down Tmod1 expression further reduced cell adhesion (Fig. 4r). Collectively, our data suggest that Tmod3, in collaboration with CDC6, might play a critical role in the drug resistance of paclitaxel.

### Tmod3 and CDC6 regulate the formation of actin stress fibers

**Fig. 5:**
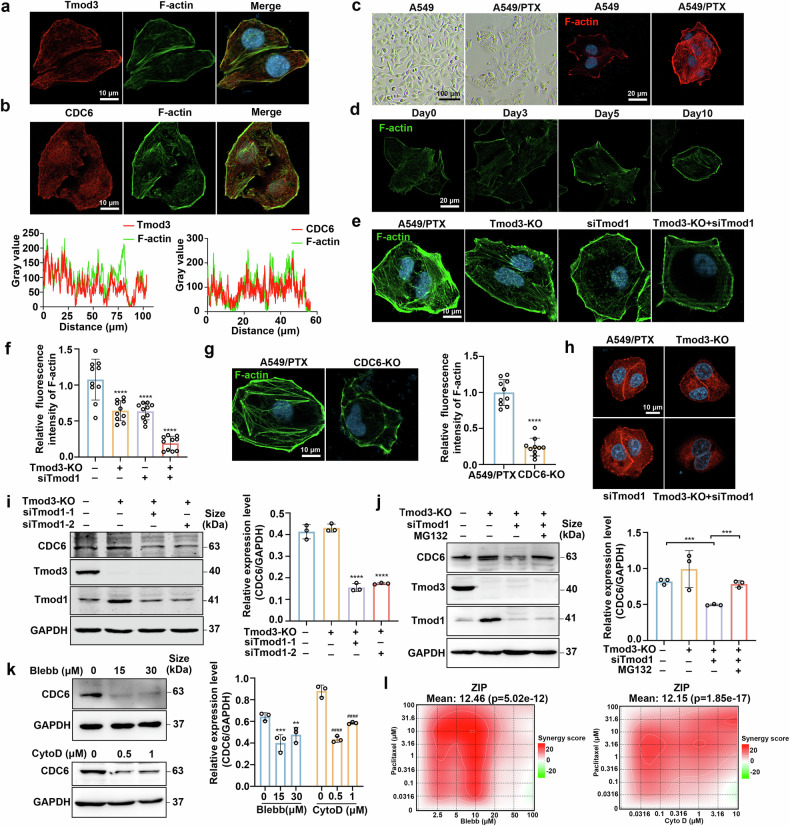
Tmod3 and CDC6 regulate the formation of actin stress fibers. **a** Colocalization of Tmod3 (red) and F-actin (green) visualized via confocal microscopy. Scale bar, 10 μm. **b** Colocalization of CDC6 (red) and F-actin (green) visualized via confocal microscopy. Scale bar, 10 μm. **c** Morphological phenotypes of sensitive and resistant A549 cell lines. Scale bars, 100 μm. Phalloidin-stained actin filaments are shown with fluorescence micrographs. Scale bar, 20 μm. **d** Confocal fluorescence micrographs showing actin filaments in A549 cells treated with paclitaxel (10 nM) for the indicated times. Scale bar, 20 µm. **e**, **f** Confocal fluorescence micrographs showing actin filaments in A549/PTX cells, Tmod1- or Tmod3-depleted cells, Tmod3- and Tmod1-depleted cells (n = 10 images; ****P < 0.0001). Scale bar, 10 µm. **g** Actin filaments in A549/PTX cells and CDC6-KO cells visualized via confocal microscopy (n = 10 images; ****P < 0.0001). Scale bar, 10 µm. **h** Confocal fluorescence micrographs of CDC6. Scale bar, 10 µm. **i** Western blotting of A549/PTX cells, Tmod3-depleted cells and Tmod3- and Tmod1-depleted cells (n = 3; ****P < 0.0001). **j** Western blotting analysis of CDC6 in Tmod3- and Tmod1-depleted cells after exposure to MG132 (2 μM) for 24 h (n = 3; ***P < 0.001). **k** Western blotting analysis of the expression of CDC6 after exposure to different doses of Blebb or CytoD for 72 h (n = 3; **P < 0.01, ***P < 0.001, ^####^P < 0.0001). **l** A549/PTX cells were exposed to paclitaxel, Blebb, CytoD and the combination of paclitaxel and Blebb or CytoD for 72 h. Cell viability was determined via the MTT assay, and ZIP scores were analyzed via Synergyfinder. The data are presented as the means ± SDs

Considering the role of Tmod3 in the elongation and depolymerization of actin filaments, we first examined its spatial relationship with actin networks. Confocal microscopy experiments confirmed that Tmod3 colocalized with stress fibers in the cytoplasm and lamellipodia at the cell edge (Fig. 5a). In addition, Tmod3 colocalized with the actomyosin ring and centrosomes in the process of mitosis, suggesting that Tmod3 may have a potential effect on mitotic functions (Supplementary Fig. 5a). Since CDC6 and Tmod3 interact with each other, we hypothesized that CDC6 may colocalize with actin, which was also proven via confocal experiments (Fig. 5b). The actin cytoskeleton is widely recognized to participate in the development of cell morphology. Therefore, we observed morphological differences between resistant and sensitive cells, and the results demonstrated enhanced spreading capacity and basal stress fiber/lamellipodia formation in paclitaxel-resistant A549/PTX cells compared with their sensitive counterparts (Fig. 5c).

Studies have shown that the structure and tension of the actin cytoskeleton are major upstream factors that regulate Yes-associated protein 1 (YAP) activity.^36^ Therefore, we investigated YAP localization, and the results revealed increased nuclear localization of YAP in A549/PTX cells compared with sensitive cells (Supplementary Fig. 5b, c). Next, we monitored the time-course of actin cytoskeleton remodeling and the distribution of YAP following continuous paclitaxel treatment in sensitive A549 cells. The results showed that prolonged treatment induced robust stress fiber formation and significantly increased YAP localization in the nucleus (Fig. 5d and Supplementary Fig. 5d), establishing cytoskeletal remodeling as a drug-adaptive mechanism. However, Tmod3 ablation alone failed to disrupt stress fiber integrity. Considering the critical effects of Tmod3 and Tmod1 in the maintenance of actin filaments,^37^ we knocked down the expression of Tmod1 via small interfering RNA (siRNA) in Tmod3-depleted cells and found almost complete disruption of stress fibers (Fig. 5e, f). Interestingly, a similar stress fiber phenotype was also detected in CDC6 knockout cells (Fig. 5g).

On the basis of the results of these experiments, we speculated that CDC6 might be carried to the cytoplasm and cell edge from the nucleus by Tmod3. However, we observed a notable reduction in CDC6 fluorescence in Tmod3- and Tmod1-deficient cells via an immunofluorescence assay (Fig. 5h), which suggested that the presence of Tmod3 and Tmod1 might influence the stability of CDC6. Next, we examined the expression level of CDC6, and the results indicated that knocking out Tmod3 alone had little effect on CDC6. In contrast, the expression of CDC6 markedly decreased in A549/PTX cells depleted of both Tmod3 and Tmod1 (Fig. 5i), which was also detected in A549 cells (Supplementary Fig. 6a). Consistent with these results, overexpression of EGFP-CDC6 in HeLa cells produced strong green fluorescence, which was considerably diminished in Tmod3- and Tmod1-depleted cells (Supplementary Fig. 6b), and western blot analysis confirmed these results (Supplementary Fig. 6c). Nevertheless, the mRNA level of CDC6 remained unaffected (Supplementary Fig. 6d). The ubiquitin-proteasome and autophagy-lysosome pathways are two primary mechanisms for the degradation of proteins.^38^ We treated Tmod3- and Tmod1-depleted cells with bafilomycin A1 (BafA1, a lysosome inhibitor) and MG132 (a proteasome inhibitor), respectively, to determine which pathway was responsible for the degradation of CDC6. The results revealed that the downregulation of CDC6 was notably rescued following MG132 treatment (Fig. 5j and Supplementary Fig. 6e), indicating that CDC6 was degraded through the proteasome pathway.

Blebbistatin (Blebb) and cytochalasin D (CytoD) are two compounds that modulate the physiological functions of actin by affecting actin contraction and actin polymerization, respectively.^39,40^ Therefore, we tested whether Belbb or CytoD treatment had an effect on CDC6, and western blotting indicated that both compounds considerably downregulated the expression of CDC6 (Fig. 5k). In addition, treatment with paclitaxel combined with either Blebb or CytoD resulted in notable synergetic effects on A549/PTX cells (Fig. 5l and Supplementary Fig. 6f, g). Thus, disrupting actin filaments might represent a promising strategy to increase the therapeutic efficacy of paclitaxel.

### Tmod3 and CDC6 accelerate focal adhesion assembly

**Fig. 6:**
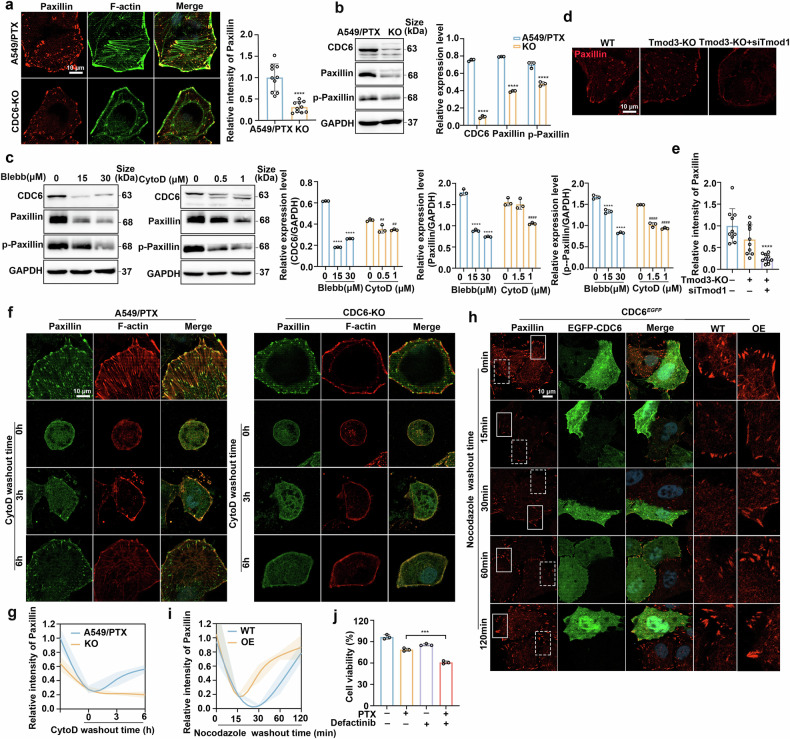
Tmod3 and CDC6 accelerate focal adhesion assembly. **a** Paxillin and actin filaments in wild type cells and CDC6-KO cells (n = 10 images; ****P < 0.0001). Scale bar, 10 µm. **b** Changes in the protein expression of CDC6, paxillin and p-paxillin in A549/PTX wild-type and CDC6-KO cells (n = 3; ****P < 0.0001). **c** Changes in the protein expression of CDC6, paxillin and p-paxillin after treatment with different concentrations of Blebb and CytoD for 72 h (n = 3; ****P < 0.0001, ^##^P < 0.01, ^####^P < 0.0001). **d**, **e** Paxillin in Tmod3- and Tmod1-depleted cells was visualized via confocal microscopy (n = 10 images; ****P < 0.0001). Scale bar, 10 µm. **f**, **g** Wild-type and CDC6-KO cells were exposed to CytoD (5 μM) for 3 h. The cells were fixed at the indicated time points after CytoD washout, stained with phalloidin and paxillin, and visualized via confocal microscopy. Scar bar, 10 μm. **h**, **i** Immunofluorescence analysis of paxillin in HeLa/CDC6^EGFP^ cells after nocodazole (10 μM) treatment for 4 h which were then washed out for the indicated times. Scale bar, 10 μm. Wild-type cells (dotted line) and CDC6^EGFP^-overexpression cells (solid line) are magnified. **j** A549/PTX cells were exposed to paclitaxel, defactinib or a combination of paclitaxel and defactinib for 72 h. Cell viability was determined via the MTT assay (n = 3; ***P < 0.001). The data are presented as the means ± SDs

The interaction between stress fibers and focal adhesions is critical for various cellular processes, including cellular structural maintenance, extracellular matrix (ECM) adhesion, cell differentiation and cell motility.^41^ Considering the influence of CDC6 on F-actin, we then detected the effect of CDC6 on cell adhesion. Immunofluorescence quantification revealed a marked reduction in the number of paxillin-positive FA puncta in CDC6-depleted cells (Fig. 6a). In addition, CDC6 depletion in both A549 and A549/PTX cells resulted in decreased expression of paxillin and phospho-paxillin (Fig. 6b and Supplementary Fig. 7a), which was reversed by CDC6 rescue experiments (Supplementary Fig. 7b). Moreover, paclitaxel treatment similarly decreased Paxillin levels (Supplementary Fig. 7c), whereas prolonged exposure led to significant Paxillin upregulation (Supplementary Fig. 7d). The observed discrepancy may be because sustained low-concentration paclitaxel treatment potentially mimics the development of drug resistance, consequently inducing paxillin upregulation as an adaptive cellular response. Pharmacological treatment (Blebb/CytoD) similarly suppressed paxillin signaling (Fig. 6c), and dual Tmod3/Tmod1 ablation also led to the disruption of focal adhesion (Fig. 6d, e), collectively implicating CDC6 as a modulator of adhesion complex integrity.

Focal adhesions that regulate multiple cellular behaviors are dynamic transmembrane macromolecular assemblies.^42^ Therefore, we treated wild-type and CDC6-KO cells with CytoD and allowed the cells to recover by washing out CytoD while we detected focal adhesion assembly at the indicated time points. The data suggested that depletion of CDC6 notably hindered FA assembly (Fig. 6f, g). Next, we constructed a nocodazole model system to further investigate the role of CDC6 in FA assembly. Nocodazole promotes microtubule depolymerization, which in turn facilitates FA assembly.^43^ Therefore, the microtubules were repolymerized, and the FAs were disassembled after washing out nocodazole. With the extension of time, the FAs then reassembled and facilitated observation. Our findings suggested that the level of paxillin strongly decreased when the cells were transfected with CDC6-EGFP or not at 15 min after nocodazole washout. Interestingly, paxillin reformation was detected in CDC6-EGFP cells after washing out nocodazole for 60 min (Fig. 6h, i), which suggested that CDC6 accelerated FA assembly in these cells. These results strongly suggest that CDC6 is required for accelerating FA assembly. Compared with paclitaxel alone, treatment with paclitaxel combined with the focal adhesion kinase inhibitor defactinib resulted in notably greater growth inhibition in A549/PTX cells (Fig. 6j).

### Blebb enhances the therapeutic effect of paclitaxel in vivo

**Fig. 7:**
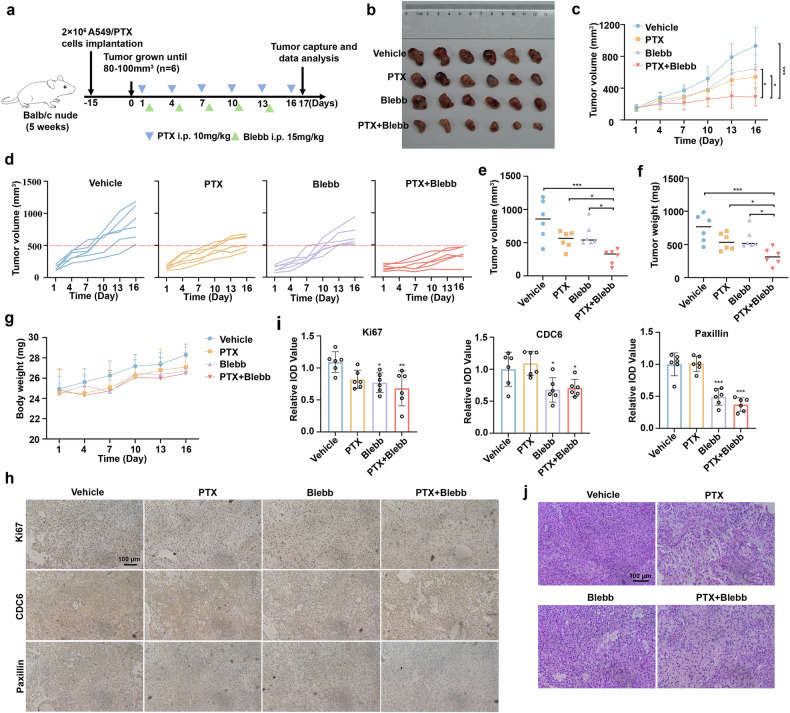
Blebb enhances the therapeutic effect of paclitaxel in vivo. **a** Establishment and drug treatment of A549/PTX xenograft mice. We injected A549/PTX cells into the right subcutaneous area of BALB/c nude mice. The colored triangles represent the compounds administered to mice. **b** Tumor images of A549/PTX xenograft mice. **c**–**e** The volume of tumors was recorded every 3 days (n = 6 mice/group; *P < 0.05, ***P < 0.001). **f** The weight of xenograft tumors after dissection (n = 6 mice/group; *P < 0.05, ***P < 0.001). **g** Mouse body weight was measured every 3 days. **h**, **i** Immunohistochemistry of CDC6 and paxillin in A549/PTX xenografts (n = 6 mice/group; *P < 0.05, **P < 0.01, ***P < 0.001). Scale bar, 100 μm. **j** H&E staining of the tumor sections. Scale bar, 100 μm. The data are presented as the means ± SDs

Emerging evidence highlights a functional link between focal adhesion signaling and chemoresistance acquisition in malignancies.^44^ Actin-myosin contractility modulates the assembly of focal adhesions and the formation of stress fibers. Given that Blebb works as a potent and specific inhibitor of myosin II^39^ and that our research demonstrated the suppressive effect of Blebb on focal adhesion, we assessed the therapeutic efficacy of paclitaxel in combination with Blebb in vivo. An A549/PTX xenograft model was used, and when the tumors reached approximately 100 mm^3^, we randomly divided the mice into four groups and treated them with vehicle, PTX (10 mg/kg), Blebb (15 mg/kg), or a combination of PTX and Blebb for 16 days. The tumor sizes were recorded, and the weights of the xenografts were measured after the mice were sacrificed (Fig. 7a). The results suggested that the tumor volume and tumor weight were notably lower in the Blebb+PTX group than in the PTX alone group (Fig. 7b–f). Furthermore, we observed no significant difference in body weight between the mice in the PTX group and those in the combined treatment group (Fig. 7g). Immunohistochemical analysis confirmed that compared with PTX alone, the combination of PTX and Blebb inhibited CDC6 and paxillin expression (Fig. 7h, i). Histopathological examination of the hematoxylin and eosin (H&E)-stained slides revealed reduced tumor aggressiveness with the combination of paclitaxel and Blebb (Fig. 7j). Moreover, the combined treatment resulted in no significant toxicity to normal tissues or immune cells (Supplementary Fig. 7e, f). In conclusion, these findings are consistent with our in vitro data, demonstrating that pharmacological targeting of CDC6-mediated adhesion signaling resensitizes resistant tumors to paclitaxel cytotoxicity.

## Discussion

Paclitaxel, the main type of microtubule stabilizing drug, is widely used in the clinic for the treatment of various cancers. However, not all patients benefit from paclitaxel and most develop drug resistance over the course of treatment.^45^ Thus far, reversing resistance to paclitaxel is still considered a challenge. Cell adhesion to the ECM generates transmembrane signals influencing cell proliferation, differentiation and survival. Among the various microenvironmental factors affecting cancer cell resistance, cell adhesion has gradually emerged as a crucial determinant, but the underlying mechanisms remain elusive.^46^ Recently, the development of CRISPR-Cas9 screening has provided a promising strategy for discovering drug resistance related genes, through which we identified CDC6 as the most critical gene associated with cell adhesion-mediated paclitaxel resistance in A549/PTX cells.

CDC6 plays a crucial role in DNA replication initiation during the G1 phase and significantly contributes to the malignant progression of various tumors.^11^ Bioinformatic analysis and experiments revealed that CDC6 was highly expressed in tumor cells, and continuous treatment of sensitive A549 cell lines with paclitaxel upregulated the expression of CDC6. The reactivities of A549/PTX cells to paclitaxel changed markedly when CDC6 expression was modulated by genetic intervention or drug treatment, as validated by xenograft studies showing that CDC6 depletion could notably sensitize A549/PTX tumors to paclitaxel. However, the inherent cytotoxicity caused by CDC6 depletion cannot be overlooked. Thus, although the critical role of CDC6 in paclitaxel resistance has been firmly established, identifying compounds that selectively target tumor cells to reduce CDC6 protein levels represents a promising therapeutic strategy.

While the conventional understanding suggests that paclitaxel exerts antitumor effects primarily through mitotic arrest, studies by Beth A. Weaver et al. revealed that the concentration of paclitaxel detected in primary breast tumors is inadequate to provoke durable mitotic arrest and that paclitaxel-induced cell death in patient tumors actually results from chromosomal missegregation caused by defective mitotic spindles.^33,47^ Research has shown that the absence of CDC6 can disrupt various biological processes.^48^ Consistent with previous studies, we found that depletion of CDC6 led to severe centrosome abnormalities and multiple divisions, increasing the possibility of chromosome missegregation and cell death. Therefore, CDC6, as a genetic regulator of multipolar division, may play a critical role in regulating paclitaxel sensitivity. Additionally, it has been reported that cancer cells can exit mitosis prematurely through slow degradation of Cyclin B, ultimately leading to drug resistance.^49^ CDC6 knockout significantly reduced the expression of p-CDK1 and upregulated Cyclin B, both of which are essential for mitotic exit through separase.^34^ Taken together, these findings suggest that targeting CDC6 may represent a promising strategy for enhancing the therapeutic efficacy of paclitaxel.

To investigate direct evidence linking CDC6 to cell adhesion, we subsequently conduced an IP assay and identified Tmod3, an actin binding protein that regulates various cellular responses, including actin remodeling, cell migration and lamellipodia formation,^17^ as an interacting partner of CDC6. Additionally, we found that Tmod3 was highly expressed in lung cancer cells and that knocking out Tmod3 enhanced the sensitivity of A549/PTX cells to paclitaxel, paralleling CDC6 depletion phenotypes. Furthermore, we observed that drug-resistant A549/PTX cells spread more extensively and that the formation of basal actin stress fibers increased in these cells. Confocal microscopy analysis suggested that Tmod3 single knockout minimally affected F-actin, whereas dual Tmod1/Tmod3 silencing eliminated stress fibers, which was recapitulated in CDC6-deficient cells. Additionally, the depletion of Tmod3 and Tmod1 led to the downregulation of CDC6, a reduction that could be reversed by the proteasome inhibitor MG132. Further investigations are expected to elucidate the precise mechanisms through which Tmod3 and Tmod1 contribute to the ubiquitin-mediated degradation of CDC6.

Stress fiber and focal adhesion formation is associated with the phosphorylation of specific proteins, including focal adhesion kinase and paxillin.^50^ Considering that cells lacking Tmod3 and Tmod1 exhibited almost complete loss of stress fibers and minimal focal adhesion,^37^ we detected focal adhesion by labeling CDC6-depleted cells with paxillin and found that the depletion of CDC6 led to a significant decrease in the expression of paxillin. Research has indicated that elevated levels of Cyclin B and the inhibition of CDK1 phosphorylation are necessary for focal adhesion disassembly.^9^ The knockdown of CDK1 notably decreased the adhesion complex area,^51^ which further demonstrated the important role of CDC6 in focal adhesion assembly. An intact actin cytoskeleton is essential for signal transduction and integrin-dependent focal adhesion assembly.^50^ Blebb and CytoD are two compounds that disrupt actin filaments by inhibiting myosin II and actin polymerization, respectively. We found that the combination of Blebb and CytoD with paclitaxel strongly enhanced the chemotherapeutic effects of paclitaxel.

The present study identified CDC6 as the gene most significantly associated with paclitaxel resistance through whole-genome CRISPR-library screening. Further investigation revealed that Tmod3 interacted with CDC6 in the cytoplasm and regulated its stability. Depletion of CDC6 resulted in the formation of abnormal centrosomes and the disruption of actin filaments. In addition, CDC6 promoted focal adhesion assembly and therefore led to paclitaxel resistance (Supplementary Fig. 8). Preclinical validation via CDC6-targeted combination therapy demonstrated superior efficacy in the treatment of xenografts without exacerbating toxicity. Interestingly, we also noted that Tmod3 was located in centrosomes, whereas CDC6 was almost completely distributed in the spindle during mitosis. It remains unclear whether the effect of CDC6 on centrosomes is dependent on the Tmod family. Further studies are needed to clarify the underlying relationship among mitosis, actin remodeling, and focal adhesion assembly regulated by the interaction of CDC6 and Tmod3. Collectively, our findings elucidate the mechanisms through which CDC6 functions as a key regulator of paclitaxel resistance and provide a potential strategy to enhance the therapeutic effects of paclitaxel.

## Materials and methods

All animal procedures were conducted in strict accordance with a protocol approved by the Animal Care and Use Committee at Shandong University. This study collected lung tissues from the human subjects and was probed by the Research Ethics Committee of the Second Hospital of Shandong University. Informed consents were obtained from the participants before the study.

### Cell lines

HEK293T, HeLa and A549 cells were cultured in DMEM. A549/PTX cells were purchased from Wuhan Pricella Biotechnology Co.,Ltd (CL-0585). The cells were cultured in RPMI 1640 medium supplemented with 20 nM paclitaxel to maintain their drug resistance. The culture medium was supplemented with 10% fetal bovine serum, 100 U/mL penicillin G and 100 µg/mL streptomycin. The cells were cultured in an incubator (Thermo Fisher Scientific) at 37 °C with 5% CO_2_. Paclitaxel (Aladdin, P106869) was dissolved in DMSO and used at the indicated doses. Norcantharidin (Aladdin, N159736), reversine (TargetMol, T1825, 0.5 μM), AZ3146 (TargetMol, T2689, 1 μM), blebbistatin (TargetMol, T21550), cytochalasin D (Aladdin, C102396), and defactinib (TargetMol, T1996, 2 μM) were dissolved in DMSO.

### Animal studies

Five-week-old male BALB/c nude mice were obtained from Gem Pharmatech (JiangSu, China). We suspended A549/PTX tumor cells (5 × 10^6^) in sterile PBS and injected them into the right subcutaneous area of the nude mice. The mice were randomly divided into designated treatment groups when the size of the tumors was approximately 100 mm^3^. The mice received the indicated treatments for a total of 16 days. We measured the tumor volume and body weight every three days. All the mice were sacrificed, and the tumors were finely excised at the end of the experiment.

### Genome-wide CRISPR/Cas9 knockout library screen

A human CRISPR knockout library was used to identify genes that contribute to paclitaxel resistance in cancer cells. The A549/PTX cell line stably expressing Cas9 was constructed via lentiviral infection with a Cas9 coding plasmid. A549/PTX-Cas9 cells were subsequently infected with the GeCKO V2.0 library, which contains 122,417 sgRNAs. These sgRNAs target 19,050 human genes, with ten sgRNAs per gene. After 48 h of infection, puromycin (4 μg/ml, Beyotime, ST551) was used for 5 days to select the genome-edited cell pools, which were then treated with vehicle or paclitaxel for an additional 8 days at a dose and duration that was lethal to control cells. The surviving cells were collected for DNA extraction and the sgRNA sequences were amplified via PCR. After DNA gel extraction, the products were subjected to massive parallel amplicon sequencing by Novogene Technology (Beijing, China).

### Virus production and infection

HEK293T cells were transfected with 2 μg of psPAX, 1.5 μg of pMD2.G and 2.5 μg of the required plasmids with 24 μl of PEI (1 mg/ml) (Servicebio, G1802) at 80–90% confluency. We replaced the culture medium with fresh medium after the cells were cultured at 37 °C for 6–10 h. The virus-containing medium was harvested at 24 h and 48 h and then filtered with a 0.45 μm filter. Finally, the target cells were infected with the prepared virus and polybrene (1000:1, 10 mg/ml) (Beyotime, C0351) for 12 h and selected with the indicated antibiotics to generate stable cell lines.

### siRNA transfection

A total of 1.5 × 10^5^ cells were plated into six-well plates and incubated for 12 h. The cells were then transfected with 50 nM indicated siRNA via jetPRIME®—Versatile DNA/siRNA transfection reagent (Polyplus, 101000046). The siRNAs that target CDC6, DUFB3, ACBD5 and the negative control siRNA were acquired from Sangon Biotech. The sequences can be found in Supplementary Table 1.

### Quantitative real-time PCR

Total RNA was obtained via the NcmSpin Cell/Tissue Total RNA Kit (New Cell & Molecular Biotech, M5105). cDNA was reverse transcribed with HiScript II Q RT SuperMix for qPCR (Vazyme, R223). RT-PCR was performed via SYBR Green (ABclonal, RK21203), following the instructions of the Bio-Rad CFX-96 system. The primers used can be found in Supplementary Table 1.

### Plasmids

Human CDC6 and Tmod3 were obtained via PCR amplification and cloned and inserted into the pcDNA3.1 vector with the Flag-tag, HA-tag and EGFP-tag. The following enzymes and kits were used in the experimental procedures: ApexHF HS DNA Polymerase FS Master Mix (Accurate Biotechnology, AG12202), T4 DNA ligase (Accurate Biotechnology, AG11810), and ClonExpress Ultra One Step Cloning Kit V2 (Vazyme, C116). The primers used can be found in Supplementary Table 1.

### Establishment of CDC6 and Tmod3 knockout cell lines

The CRISPR/Cas9 knockout plasmid of CDC6 was obtained from Beyotime (L22060), and the sgRNA of Tmod3 was cloned and inserted into the lentiCRISPR V2 plasmid. The sequences can be found in Supplementary Table 1. HEK293T cells were used for virus production, and A549/PTX cells were selected with puromycin (4 μg/ml) for 3 days after viral transduction. Then, the monoclonal cells were selected to obtain stable knockout cell lines.

### MTT and cell morphology assays

We plated 5 × 10^3^ cells per well in 96-well plates and allowed them to adhere for 12 h. Afterwards, we exposed the cells to different concentrations of paclitaxel for 72 h, followed by the addition of MTT (Genview, JT-343) for another 4 h. DMSO was used to dissolve the generated crystals. The viability of the cells was tested at 570 nm via a microplate reader (BioTek, USA). The morphological changes in the cells were observed via an inverted microscope (Olympus BX6).

### IC_50_ determination

To determine the IC_50_ values, dose-response curves were generated via GraphPad Prism. The data were normalized to those of the vehicle-treated control group, which was set to 100% viability. In addition, we normalized the data by setting the top constraint to 100%, and the bottom plateau was constrained to a value between zero and the cell viability observed at the highest drug concentration. The dose-response curves were then generated by fitting the log-transformed concentration data to a four-parameter logistic nonlinear regression model. The IC_50_ values, 95% confidence intervals (CIs), and hill slopes were calculated from the resulting curves.

### Immunohistochemical (IHC) assay

We embedded the clinical tissue samples and tumor samples in paraffin and performed antigen retrieval. After blocking endogenous peroxidase activity, the samples were incubated with the indicated primary antibodies, followed by incubation with the appropriate secondary antibodies, as shown Supplementary Table 2. Differences in protein expression were detected via DAB (Servicebio, G1212) reagents and visualized via an inverted microscope (Olympus BX6).

### Cell proliferation and apoptosis assays

We determined cell proliferation in vitro by counting the number of cells for 3 to 5 days following treatment with paclitaxel. A total of 3 × 10^4^ cells per well were plated in a 12-well plate and exposed to paclitaxel (300 nM) or vehicle for the indicated times. The number of cells was counted via an automated cell counter (IC100) (Countstar, China). For the apoptosis assay, we seeded 2 × 10^5^ cells per well in a 6-well plate and incubated them for 12 h. The cells were exposed to paclitaxel (600 nM) and incubated for another 72 h, followed by flow cytometry analysis, which was performed via an Annexin V-FITC/PI Kit (Yeasen, 40302ES) following the protocol of the manufacturer.

### Cell cycle analysis

We plated 3 × 10^5^ cells per well in a 6-well plate and incubated them for 12 h. After treating the cells with paclitaxel (50 nM) for 24 h, we prepared the samples via a Cell Cycle and Apoptosis Analysis Kit (Beyotime, C1052) following the protocol of the manufacturer. A FACSCalibur flow cytometer (BD Biosciences, CA, USA) was used to monitor cell cycle arrest. FlowJo software was used to analyze the proportion of cells in each phase of the cell cycle.

### Clonogenic assay

We seeded 400 cells per well in a 6-well plate and replaced the culture medium containing paclitaxel (50 nM) after 2 days. Following a 10-day culture period, we fixed the cells with 4% paraformaldehyde and stained them with 0.1% crystal violet (Aladdin, C299450) for 15 min. The results were obtained and analyzed via Image J.

### Wound-healing assay

We plated 1 × 10^6^ cells per well in a six-well plate and incubated them overnight. We created a linear scratch on the cell surface via a 200 μl pipette tip and washed the cells gently with PBS to remove the unattached cells. The cells were subsequently exposed to paclitaxel, and images were obtained via an inverted microscope (Olympus BX6).

### Transwell assay

The migration assay was conducted using a 24-well Transwell chamber system (Corning, USA). The upper chambers were seeded with 4 × 10^4^ cells that were serum free, while the lower chambers were supplemented with culture medium that contained 10% FBS. After incubation for 24 h, we gently removed the nonmigrating cells with cotton swabs and fixed the migrating cells with 4% paraformaldehyde, followed by staining with crystal violet (Aladdin, C299450) for 15 min. The results were obtained via an inverted microscope (Olympus BX6).

### Immunoprecipitation assay

HEK293T cells were transfected with the CDC6-HA plasmid and lysed with western-IP lysis buffer (Beyotime, P0013). Anti-HA magnetic beads (MCE, HY-K0201) were incubated with total cell lysates and conjugated overnight in a rotating shaker. To remove the uncombined proteins, the beads were washed sufficiently with cell lysis buffer the next day. Potential proteins that may interact with CDC6 were bound to magnetic beads and boiled for 10 min. Then, the beads were removed via a magnetic separation rack. The samples were subjected to SDS-PAGE and stained with a Fast Silver Stain Kit (Beyotime, P0017S). Then we excised the differential bands in the gels, performed in-gel digestion and analyzed the samples through LC-MS/MS.

### Co-IP assay

HEK293T cells in 60 mm dishes were transfected with CDC6-HA and TMOD3-Flag plasmids as required and were subsequently lysed with western-IP lysis buffer. Anti-HA magnetic beads and anti-Flag magnetic beads (MCE, HY-K0207A) were incubated with total cell lysates and incubated overnight in a rotating shaker. To remove the uncombined proteins, the beads were washed sufficiently with cell lysis buffer on the second day and boiled for 10 min at 100 °C. The beads were then removed via a magnetic separation rack and subjected to western blotting. For endogenous Co-IP of CDC6 and Tmod3, A549 and A549/PTX cells were plated in 100 mm dishes and lysed with IP lysis buffer. Four micrograms of anti-CDC6 antibody and control IgG were incubated with total cell lysates and incubated overnight in a rotating shaker. Fifty microliters rProteinA/G beads were incubated with the lysates for additional 4 h. The elution step was performed following the instructions provided with the Immunoprecipitation Kit (Proteintech, PL10007). The results were analyzed via western blotting.

### Bimolecular fluorescence complementation (BiFC) assay

CDC6 and Tmod3 were cloned and inserted into the pBiFC-VN and pBiFC-VC vectors, respectively, to create N- and C-terminal Venus fusion constructs. HEK293T cells were co-transfected with the indicated plasmid pairs. After 48 h, live-cell images were acquired via an inverted fluorescence microscope (Zeiss Axiovert 5).

### Western blotting

Western-IP lysis buffer was used to lyse the cells to be tested, and the samples were centrifuged to obtain the supernatant. The protein concentrations of the samples were determined with a BCA assay kit (Elabscience, E-BC-K318-M). We separated equal amounts of proteins via SDS-PAGE and transferred them onto nitrocellulose membranes with a constant current. The membranes were blocked with 5% nonfat milk in TBST buffer (Servicebio, G2150) for 1 h at room temperature. The indicated primary antibodies, which were listed in Supplementary Table 2, were subsequently incubated with the membranes overnight at 4 °C. The following day, the membranes were washed with TBST three times and incubated with the indicated secondary antibodies at room temperature for 1 h. The bands were visualized via SuperSignal SuperDura Extended Duration Substrate (Yeasen, 36208ES) and Fluorescence Imager (Amersham Imager 600 RGB, Japan).

### Immunofluorescence

The confocal dishes were seeded with 1 × 10^5^ cells and cultured overnight. Then, 4% paraformaldehyde was applied to fix the cells for 10 min, and 0.1% Triton X-100 was used to permeabilize the samples at room temperature for 15 min. We blocked the fixed cells with 3% BSA (Solarbio, A8020), and the indicated primary antibodies were used to incubate with the samples overnight at 4 °C. On the following day, the cells were incubated with TBST three times and incubated with the indicated secondary antibodies at room temperature for 1 h. Then the samples were stained with DAPI (Beyotime, C1002) for 15 min. Images were obtained with a Zeiss LSM900 confocal laser scanning microscope (Carl Zeiss, Germany).

### Time-lapse imaging of cells

A 96-well plate was seeded with 4 × 10^3^ cells per well and cultured overnight. Then time-lapse imaging was conducted using an ImageXpress Micro 4 (Molecular Devices, USA).

### Nocodazole model system

The confocal dishes were seeded with 1 × 10^5^ HeLa cells that were transiently transfected with the CDC6-EGFP plasmid and cultured for 48 h. The cells were exposed to 10 μM nocodazole (TargetMol, T2802) for 4 h to depolymerize the microtubes completely. Then, serum-free medium was used to wash out the nocodazole. In addition, 4% paraformaldehyde was applied to fix the cells at the indicated times for 10 min. IF staining was performed with paxillin antibody. Images were obtained with a Zeiss LSM900 confocal laser scanning microscope (Carl Zeiss, Germany).

### Cell adhesion assay

Fibronectin (BD Biosciences, USA) was used to coat 24-well plates for 1 h at 37 °C. After the plates were blocked with 1% bovine serum albumin, 5 × 10^4^ cells were plated in each well and cultured for 30 min at 37 °C. Then, 4% paraformaldehyde was applied to fix the cells, which were then stained with 0.1% crystal violet. Images were obtained via an inverted microscope (Olympus BX6).

### Quantification and statistical analysis

The data are presented as the means ± SDs for three independent experiments. Differences between groups were analyzed by two-tailed unpaired Student’s *t*-test. Multiple group comparisons were analyzed via one-way ANOVA followed by the Tukey post-hoc test with GraphPad Prism 9.5 (San Diego, CA), and a p- value less than 0.05 was considered statistically significant.

## Supplementary information

Original western blots

Supplementary Materials

## Data Availability

The genome-wide CRISPR/Cas9 knockout screening data were deposited in the SRA database with the accession number PRJNA1299550, and the RNA-sequencing data of A549 and A549/PTX cells were deposited in the GEO database with the accession number GSE304051.
