# Supplementary figures and images for: Interaction between CDC6 and Tmod3 accelerates resistance to paclitaxel through focal adhesion assembly
Source: Signal Transduct Target Ther. 2025 Dec 4;10:395. doi: 10.1038/s41392-025-02490-7 (PMC12675664; doi:10.1038/s41392-025-02490-7)

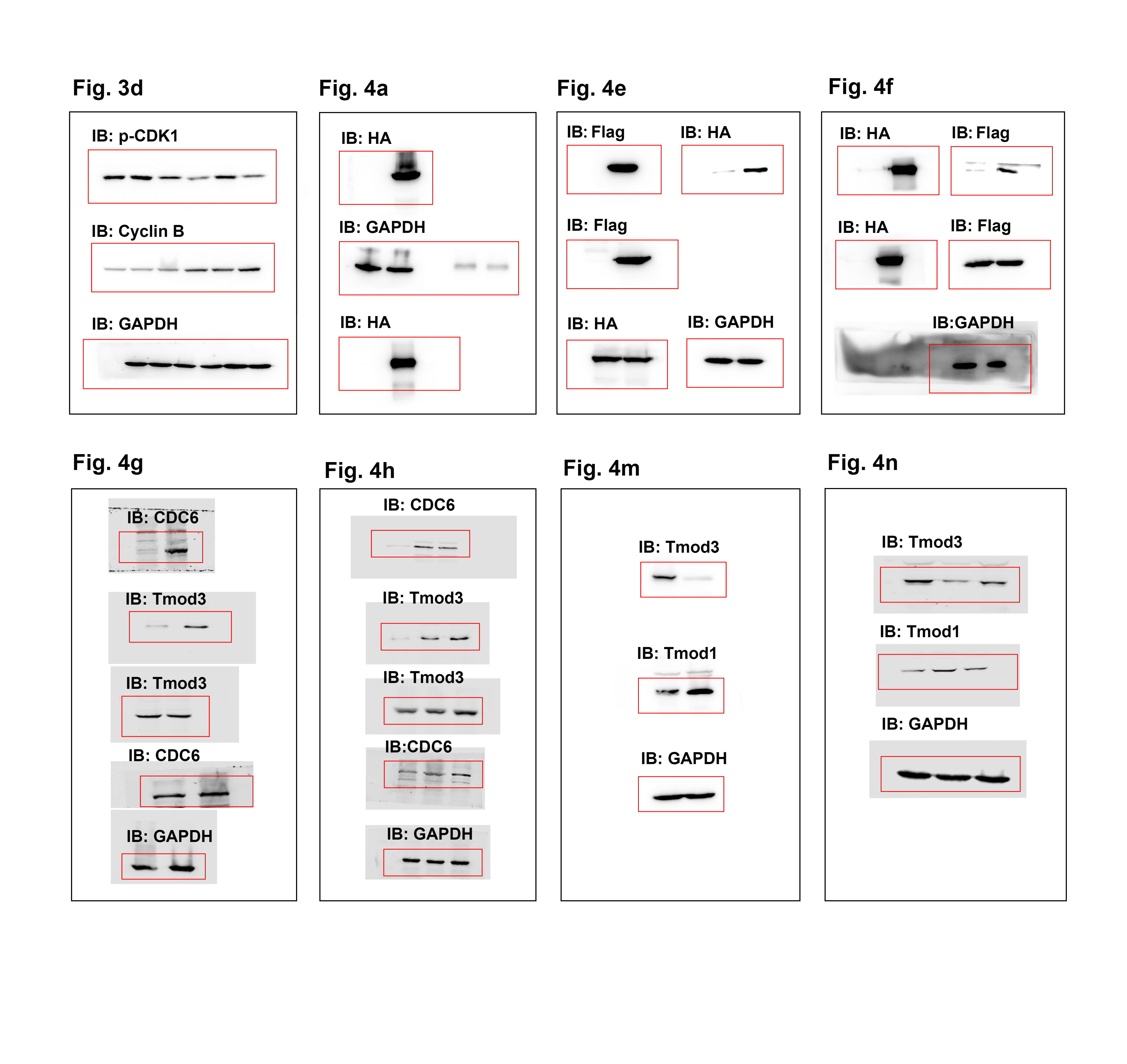

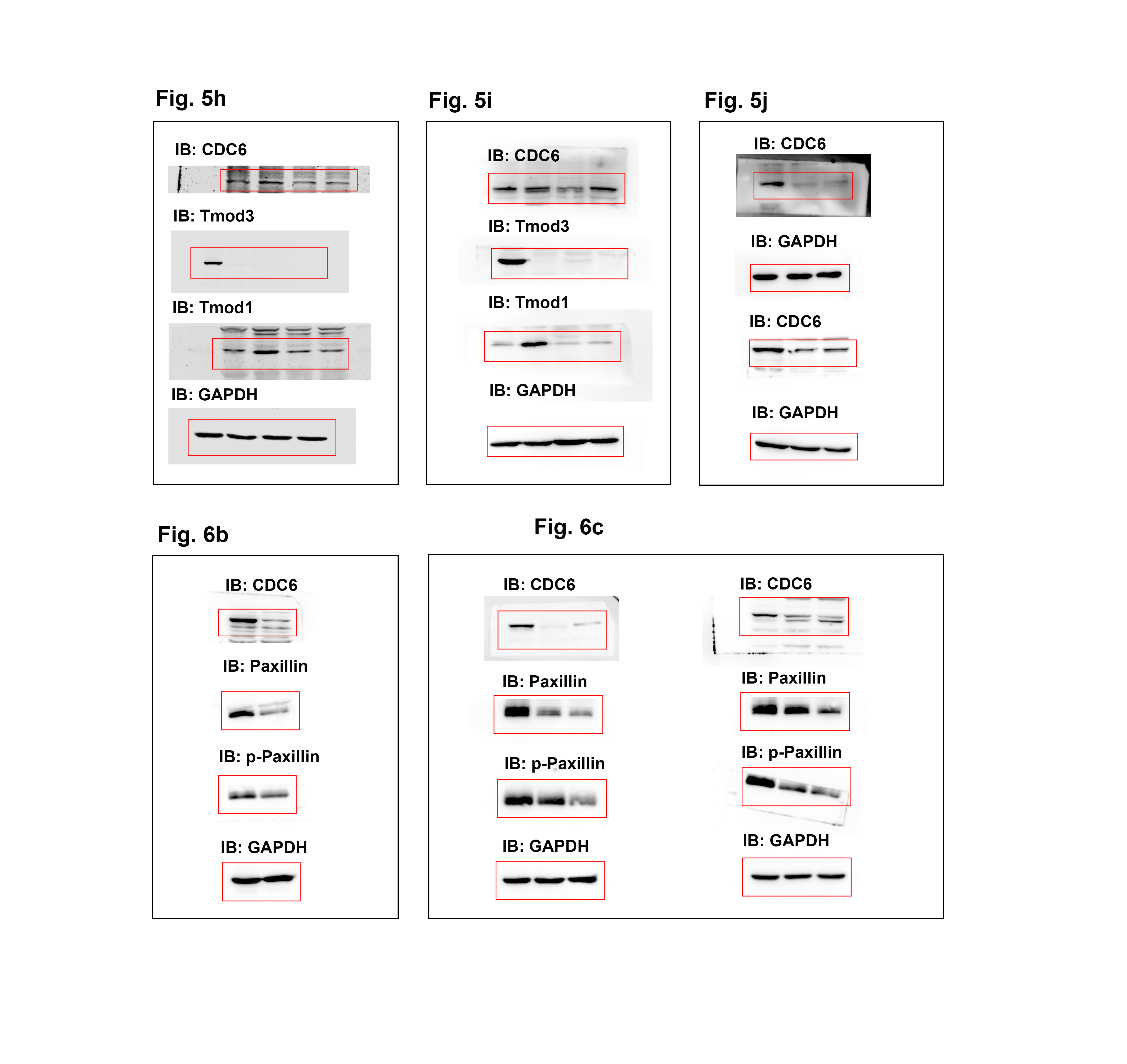

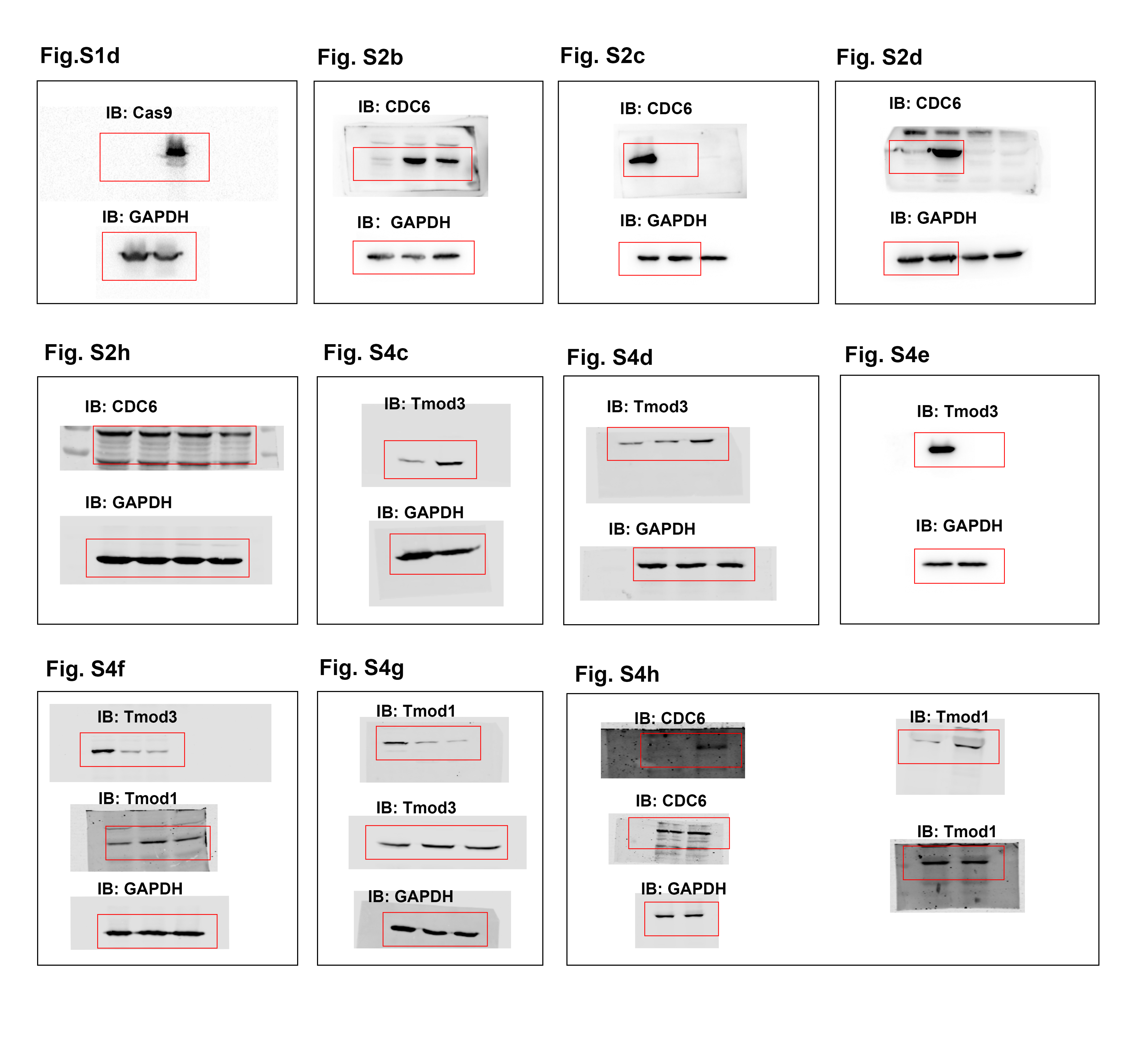


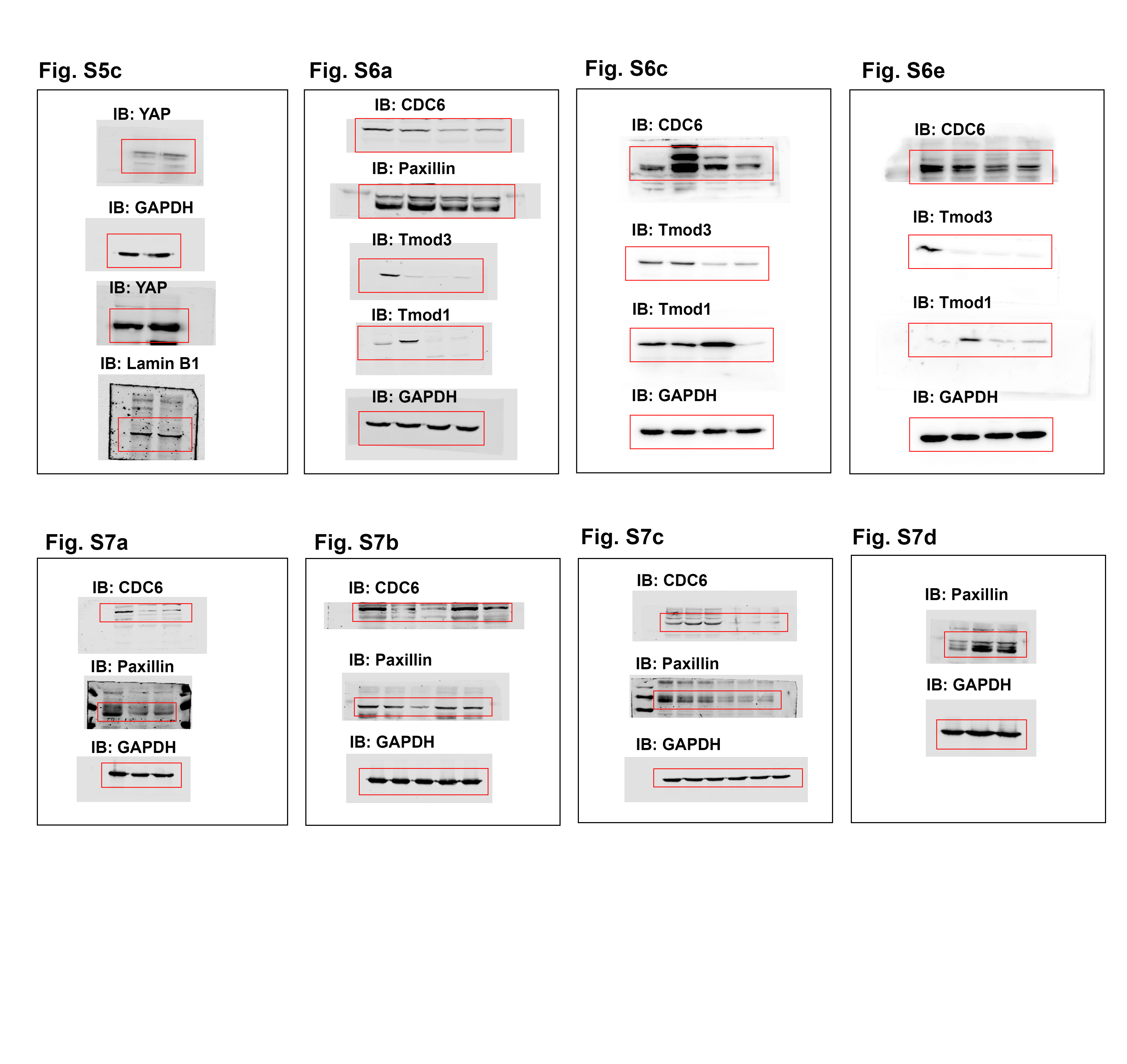

Supplement: Supplementary file 1 — Original western blots [file 41392_2025_2490_MOESM1_ESM.docx]
