# Supplementary material for: Interaction between CDC6 and Tmod3 accelerates resistance to paclitaxel through focal adhesion assembly
Source: Signal Transduct Target Ther. 2025 Dec 4;10:395. doi: 10.1038/s41392-025-02490-7 (PMC12675664; doi:10.1038/s41392-025-02490-7)
Supplement: Supplementary file 2 — Supplementary Materials [file 41392_2025_2490_MOESM2_ESM.docx]

Supplementary Materials for

Interaction between CDC6 and Tmod3 accelerates resistance to paclitaxel through focal adhesion assembly

Yue Liu, Huirui Wang, Jie Zhan, Jiabo Sun, Yan Sun, Xiaojie Fu, Dongxue Lv, Xiuyun Li, Ting Dong, Hongxiang Lou

Correspondence to: [louhongxiang@sdu.edu.cn](mailto:louhongxiang@sdu.edu.cn), [tingdong2021@sdu.edu.cn](mailto:tingdong2021@sdu.edu.cn)

**This PDF file includes:**

Supplementary Figures S1 to S7

Supplementary Tables S1 to S2

**Figure S1.**

**Figure S1. CRISPR library screening identified CDC6 as a driver for paclitaxel resistance.** (a) Volcano map showing the genetic differences between paclitaxel sensitive and resistance A549 cells. (b) KEGG analysis of the enriched differential genes and focal adhesion pathway. (c) Heatmap showing significantly changed focal adhesion-related gene expression levels identified by RNA-sequencing analysis, which compared parental and resistant cells. (d) Western blotting analysis of the stable overexpression of Cas9 in A549/PTX cells. (e) Parental and paclitaxel resistance cells of A549 were exposed to different doses of paclitaxel for 72 h. MTT assay was used to detect cell viability. (f) Quantitative real-time PCR analysis was used to detect the mRNA amount of NDUFB3 and ACBD5 after A549/PTX cells were transfected with small-interfering RNA (n=3; ***P<0.001, ****P<0.0001). (g) A549/PTX cells were exposed to paclitaxel for 72 h after knocked down the expression of NDUFB3 or ACBD5, and MTT assay was used to detect cell viability. The data are presented as the means ± SDs.

**Figure S2.**

**Figure S2. Inhibition of CDC6 sensitized A549/PTX cells to paclitaxel treatment.** (a) mRNA expression levels of CDC6 in tumor tissues and normal tissues. Data were shown as box-and-whisker plots (*P<0.05). The image was obtained from the DEPIA database. (b) Western blotting analysis of the expression of CDC6 in A549 sensitive cells after treated with paclitaxel (10 nM) for 3 days and 7 days. (c) Western blotting analysis of the knockout of CDC6 in A549/PTX cells. (d) Western blotting analysis of the overexpression of CDC6. (e) A549 cells and CDC6-OE cells were exposed to indicated doses of paclitaxel for 72 h. Cell viability was detected by MTT assay. (f) Images of tumors from A549/PTX xenograft mice. (g) IHC examination of Ki67 expression in tumor tissues and representative H&E staining of the tumor sections. Scale bar, 100 μm. (h) Western blotting analysis of the expression of CDC6 after treated with NCTD. (i) A549/PTX cells were exposed to paclitaxel and NCTD for 72 h and MTT assay was used to detect cell viability (n=3; ***P<0.001). (j) NCTD augmented paclitaxel induced apoptosis. (k) Colony formation assay in A549/PTX cells treated with paclitaxel and NCTD (n=3; **P<0.01). (l) Establishment and treatment of A549/PTX xenograft mice with PTX, NCTD and the combination of PTX and NCTD. The data are presented as the means ± SDs.

**Figure S3.**

**Figure S3. Quantification of abnormal centrosome and spindle rate in CDC6 deficient cells.** (a) Quantification of abnormal centrosome rate in CDC6 deficient cells (n=3; ****P<0.0001). (b) Quantification of abnormal spindle rate in CDC6 deficient cells (n=3; ***P<0.001). The data are presented as the means ± SDs.

**Figure S4.**

**Figure S4. Tmod3 interacts with CDC6 and is associated with paclitaxel resistance.** (a) Confocal images representing the colocalization of CDC6 (green) and Tmod3 (red) in A549 cells. Scale bar, 10 μm. Merged image (right panel) represented the co-localization tracer profile. (b) Bimolecular fluorescence complementation assay of CDC6 and Tmod3. Scale bar, 50 μm. (c) Western blotting analysis of the expression of Tmod3 in A549 and A549/PTX cells (n=3; ***P<0.001). (d) Western blotting analysis of the expression of Tmod3 in A549 sensitive cells after treated with paclitaxel (10 nM) for 8 days and 15 days (n=3; ****P<0.0001). (e) Western blotting analysis of the knockout of Tmod3. (f) The expression changes of Tmod1 in Tmod3-deficient A549 cells (n=3; **P<0.01, ***P<0 .001, ****P<0.0001). (g) The expression changes of Tmod3 in Tmod1-deficient A549 cells (n=3; ****P<0.0001). (h) Co-IP assay was performed in A549/PTX cells and the interactions between CDC6 and Tmod1 were analyzed using western blotting. The data are presented as the means ± SDs.

**Figure S5.**

**Figure S5. Paclitaxel-resistant A549 cells exhibit actin cytoskeleton remodeling.** (a) Representative confocal images showing the colocalization of Tmod3 (red) and F-actin (green) in the process of mitosis. Scale bar, 10 μm. (b) Immunofluorescence analysis of cells with YAP (red) antibody and DAPI (blue). Scale bar, 10 µm. The histogram showed the ratio of YAP distribution in nucleus to cytoplasm in paclitaxel resistant and sensitive cells (n=8 images; ***P<0.001). (c) Western blotting analysis of the expression of YAP in the cytoplasm and nucleus (n=3; ****P<0.0001). (d) Confocal fluorescence micrographs showing YAP distribution in A549 cells treated with paclitaxel (10 nM) for the indicated times. Scale bar, 20 µm. The data are presented as the means ± SDs.

**Figure S6.**

**Figure S6. Tmod3 and Tmod1 regulate the expression of CDC6.** (a) Western blotting assay in A549 cells, Tmod3 or Tmod3 depleted cells and Tmod3 and Tmod1-depleted cells (n=3; ****P<0.0001). (b) EGFP-CDC6 plasmids were transfected into Hela cells, Tmod3-KD cells and Tmod3 and Tmod1-KD cells. An inverted fluorescence microscope was used to capture the images. Scale bar, 200 µm. (c) Western blotting analysis of the expression of CDC6 and Tmods (n=3; *P<0.05, ****P<0.0001 related to control group; ^####^P<0.0001 related to EGFP-CDC6 group). (d) Quantitative real-time PCR analysis was used to detect the mRNA amount of CDC6 in Tmods depleted cells. (e) Western blotting analysis of CDC6 in Tmods depleted cells after treatment with BafA1 (100 nM) for 24 h (n=3; ***P<0.001). (f, g) A549/PTX cells were exposed to paclitaxel, Blebb, CytoD and the combination of paclitaxel and Blebb or CytoD for 72 h. Cell viability was detected using MTT assay. HSA, Loewe, Bliss scores were analyzed using Synergyfinder. The data are presented as the means ± SDs.

**Figure S7.**

**Figure S7. Tmod3 and CDC6 accelerate focal adhesion assembly.** (a) Western blotting analysis of Paxillin in CDC6-depleted A549 cells. (b) Western blotting analysis of Paxillin in CDC6-depleted cells and CDC6-reconstituted cells (n=3; **P<0.01, ***P<0.001). (c) Western blotting analysis of Paxillin after treatment with paclitaxel (n=3; **P<0.01, ***P<0.001). (d) Western blotting analysis of the expression of Paxillin in A549 sensitive cells after treated with paclitaxel (10 nM) for 8 days and 15 days (n=3; ****P<0.0001). (e) Representative H&E staining of the normal tissues. Scale bar, 100 µm. (f) IHC examination of CD45 expression in normal tissues. Scale bar, 100 µm. The data are presented as the means ± SDs.

**Figure S8.**

**Figure S8. Schematic representation of the mechanisms by which CDC6 and Tmod3 accelerate resistance to paclitaxel through focal adhesion assembly.**

**Table S1.**

**Oligonucleotides used in this study**

| **Primers for**  **recombinant DNA** | **Forword** | **Reverse** |
| --- | --- | --- |
| CDC6-HA | CGCGGATCCATGCCTCAAACC  CGATCCCAGGCAC | CCGCTCGAGTTAAGGCAA  TCCAGTAGCTAAGATA |
| CDC6-EGFP | CTCCACAGGTGTAAGGAATTC  ATGCCTCAAACCCGATCCC | TTCCAGCATGGTGGCCTCGAGAGCAATCCAGTAGCTAAGATATTTCC |
| TMOD3-Flag | CGCGGATCCATGGCACTGCCATTCCGTAAGGA | CCGCTCGAGTTACTGGTGATCTCCTTCAACTCG |
| TMOD3-EGFP | CCGGAATTCATGGCACTGCCA  TTCCGTAAGGA | CCGCTCGAGCTGGTGATCTCC  TTCAACTCG |
| **Primers for qPCR** |  |  |
| NDUFB3 | GCTGGCTGCAAAAGGGCTA | CTCCTACAGCTACCACAAATGC |
| ACBD5 | GCACGAGACTAGGTTTGAGGC | GTGAACTCCAAGCATCCCATTTA |
| CDC6 | CCAGGCACAGGCTACAATCAG | AACAGGTTACGGTTTGGACATT |
| β-actin | GATCATTGCTCCTCCTGAGC | ACTCCTGCTTGCTGATCCAC |
| **siRNA sequence** | **sense (5'-3')** | **antisense (5'-3')** |
| NDUFB3 | UGGCUUUGCAAAGAGUGUUUC | GAAACACUCUUUGCAAAGCCA |
| ACBD5 | GCACAGUGGUUGGUGUAUUUA | UAAAUACACCAACCACUGUGC |
| CDC6-1 | AACUUCCCACCUUAUACCAGA | UCUGGUAUAAGGUGGGAAGUU |
| CDC6-2 | AAGAAUCUGCAUGUGUGAGAC | GUCUCACACAUGCAGAUUCUU |
| Tmod3-1 | ACAAUGACUUAGUGCGUAA | UUACGCACUAAGUCAUUGU |
| Tmod3-2 | GCGUUAAGAGAUAAUGAAA | UUUCAUUAUCUCUUAACGC |
| Tmod1-1 | CCUCAAUAAUAUCCGGAAUTT | AUUCCGGAUAUUAUUGAGGTT |
| Tmod1-2 | GUAAUGACCCCGUGGCGUA | UACGCCACGGGGUCAUUAC |
| **sgRNA sequence** |  |  |
| CDC6 | CACCGTGTGCCTGGGATCGGGTTTG | AAACCAAACCCGATCCCAGGCACAC |
| TMOD3 | CACCGCAAACAGAAACCTGTACAGA | AAACTCTGTACAGGTTTCTGTTTGC |

**Table S2.**

**Antibodies used in this study**

| **Antibodies** | **Source** | **Identifier** |
| --- | --- | --- |
| Cas9 rabbit mAb | Abcam | Cat#ab203933 |
| Pericentrin mouse mAb | Abcam | Cat#ab28144 |
| CDC6 rabbit pAb | Proteintech Group | Cat#11640-1-AP |
| GAPDH mouse mAb | Proteintech Group | Cat#60004-1-Ig |
| TMOD3 rabbit pAb | Proteintech Group | Cat#12521-1-AP |
| Paxillin rabbit pAb | Proteintech Group | Cat#10029-1-Ig |
| TMOD1 rabbit pAb | Proteintech Group | Cat#10145-1-AP |
| HA mouse mAb | Abmart | Cat#M20021 |
| Flag mouse mAb | Abmart | Cat#M20008 |
| p-CDK1 rabbit mAb | Abways | Cat#CY5304 |
| Cyclin B rabbit mAb | Abways | Cat#CY5531 |
| YAP rabbit mAb | Abways | Cat#CY5381 |
| p-Paxillin | Abways | Cat#CY5891 |
| α-tubulin mouse mAb | ABclonal Technology | Cat#AC012 |
| HRP Rabbit Anti-Goat IgG (H+L) | ABclonal Technology | Cat#AS029 |
| HRP Mouse Anti-Goat IgG (H+L) | ABclonal Technology | Cat#AS003 |
| Rhodamine Goat Anti-Rabbit IgG (H+L) | ABclonal Technology | Cat# AS040 |
| FITC Goat Anti-Rabbit IgG (H+L) | ABclonal Technology | Cat# AS011 |
